# NUTM2A-AS1 as a potential key regulator in cancer: unraveling its ceRNA networks and impact on tumor biology

**DOI:** 10.1186/s40001-025-03019-y

**Published:** 2025-09-03

**Authors:** Negar Taghavi Pourianazar, Safa Radmehr, Zahra Ourang, Kaveh Jaseb, Alireza Asadi

**Affiliations:** 1 Medical Laboratory Techniques, Istanbul Aydin University, Istanbul, Turkey; 2 Thalassemia & Hemoglobinopathy Research Center/ Health Research Institute/Ahvaz, , Ahvaz, Iran; 3 Arak University of Medical Sciences, Arak, Iran; 4 Dentistry School, Shiraz University of Medical Sciences, Shiraz, Iran

**Keywords:** Neoplasms, ceRNA, Long noncoding RNAs, NUTM2A-AS1, Biomarker

## Abstract

NUTM2A-AS1 is an emerging long noncoding RNA (lncRNA) that has garnered significant attention due to its multifaceted roles in cancer biology. As a member of the ceRNA network, NUTM2A-AS1 modulates gene expression by sequestering microRNAs, thereby influencing key oncogenic pathways. This review aims to provide a comprehensive overview of the current understanding of NUTM2A-AS1 in the development, progression, and metastasis of various cancers, including gastric cancer, hepatocellular carcinoma, neuroblastoma, colorectal cancer, glioma, lung adenocarcinoma, prostate cancer, and renal cell carcinoma. A systematic evaluation of experimental, clinical, and bioinformatics studies was conducted, with an emphasis on studies reporting expression patterns, mechanistic insights, and clinical correlations. Key findings reveal that in gastric cancer, NUTM2A-AS1 functions as a ceRNA for miR‑376a, leading to upregulation of TET1 and HIF-1A and subsequent increase in PD-L1 expression, while also modulating *matrine* resistance via the miR‑613/ROS/VEGFA axis. In hepatocellular carcinoma, it sponges miR‑186‑5p, thereby derepressing KLF7 and activating the Wnt/β catenin pathway. Neuroblastoma studies demonstrate that NUTM2A-AS1 enhances chemoresistance and metastasis through stabilization of B7-H3, mediated by NR1D1. In colorectal cancer, its transcriptional activation by H3K27 acetylation enables it to sequester miR-126-5p and upregulate FAM3C. Similar ceRNA-driven mechanisms involving miR-376a-3p/YAP1 in glioma, miR-590-5p/METTL3 in lung adenocarcinoma, and miR-376a-3p/PRMT5 in prostate cancer further underscore its oncogenic potential. In addition, NUTM2A-AS1 is incorporated into prognostic lncRNA signatures for renal cell carcinoma. The clinical implications of these findings are significant, as NUTM2A-AS1 holds promise as a biomarker for cancer diagnosis and prognosis and as a target for novel therapeutic strategies. Future research should prioritize in vivo studies and clinical trials, leveraging emerging technologies such as CRISPR and single-cell RNA sequencing, to fully elucidate the therapeutic potential of targeting NUTM2A-AS1 in personalized cancer treatment.

## Background

Cancer has been recognized as the second-leading cause of death globally, with its burden manifested through millions of fatalities and an enormous impact on public health, society, and economies [1]. In 2020, cancer was implicated in approximately 5.5 million deaths among men and 4.4 million among women [2]. The devastating toll of the disease has been observed not only in direct mortality but also in the broader societal repercussions, including the emotional and economic distress experienced by patients, families, and health systems [3, 4]. Moreover, the indirect consequences, such as the increased number of orphans resulting from cancer-related parental deaths, have underscored the far-reaching impact of this disease, particularly in regions such as sub-Saharan Africa where breast cancer mortality has left numerous children parentless [5]. Cancer has been attributed to nearly one in six deaths worldwide and is responsible for a significant proportion of non-communicable disease mortality, especially among individuals aged 30–69 years [6]. The disease’s pervasive influence has been shown to limit life expectancy while inflicting variable societal and macroeconomic costs across different cancer types, geographical regions, and genders [6].

The human genome is composed of roughly 20,000 protein-coding genes, which account for less than 2% of its entirety, while the remaining majority was once dismissed as “junk DNA [7–9].” However, advances in high-throughput technologies such as deep RNA sequencing have revealed the existence of thousands of transcripts that do not code for proteins [10]. These noncoding RNAs (ncRNAs) have since emerged as key regulators in various biological processes, and their roles in oncogenesis have become the subject of intensive study [11]. ncRNAs are broadly divided into housekeeping and regulatory categories [12]. The housekeeping group comprises molecules such as transfer RNAs, small nuclear RNAs, small nucleolar RNAs, ribosomal RNAs, and related fragments, which are essential for basic cellular functions [11]. In contrast, regulatory ncRNAs are further classified into circular RNAs and linear RNAs; the latter are subdivided by size into small noncoding RNAs, typically under 200 nucleotides, and long noncoding RNAs (lncRNAs), which exceed this length [13, 14]. The small RNA subgroup includes microRNAs, small interfering RNAs, Piwi-interacting RNAs, and enhancer RNAs [15, 16]. The significance of these ncRNAs has been increasingly recognized, particularly in the context of cancer, where they are implicated in the modulation of gene expression and represent promising targets for both diagnosis and therapeutic intervention.

Long noncoding RNAs (lncRNAs) have been identified as pivotal regulators of gene expression, exerting influence over numerous cellular processes and disease states [17, 18]. Defined as RNA transcripts exceeding 200 nucleotides in length that lack protein-coding potential, lncRNAs regulate gene expression through a variety of mechanisms including chromatin remodeling, transcriptional modulation, and post-transcriptional processing [7]. In several cellular contexts, lncRNAs have been observed to control critical biological events such as cell proliferation, differentiation, and apoptosis [19, 20]. Their regulatory versatility has positioned lncRNAs as attractive candidates for novel therapeutic approaches and as potential biomarkers for disease diagnosis and prognosis [19, 20]. In addition, lncRNAs are known to function as competing endogenous RNAs (ceRNAs), whereby they sequester microRNAs and thereby indirectly modulate the expression of target mRNAs [21–23]. The ceRNA networks formed by lncRNAs have been increasingly implicated in the initiation and progression of multiple cancer types.

Numerous long noncoding RNAs have been reported to exhibit aberrant expression in cancer, with some, such as GAS5 and HOTAIR, emerging as prominent examples [24–27]. The lncRNA GAS5 has been widely documented as being significantly downregulated in various cancer types [28–30]. It has been demonstrated to interact with multiple microRNAs, thereby influencing critical signaling cascades such as the SUFU (Suppressor of Fused Homolog), Wnt/β-catenin, and PI3K/AKT pathways [31–33]. The diminished expression of GAS5 is thought to contribute to dysregulation in cellular processes including autophagy, oxidative stress response, and immune modulation, ultimately leading to poor patient prognoses and altered chemoradiation sensitivity [34, 35]. Conversely, HOTAIR has been found to be overexpressed across a broad spectrum of tumors, where it functions as an oncogenic driver by acting as a dual molecular scaffold [36–38]. This unique property enables HOTAIR to coordinate histone methylation and demethylation processes, leading to the reprogramming of chromatin states and the silencing of tumor suppressor genes [39, 40]. HOTAIR has also been implicated in promoting epithelial–mesenchymal transition, metastasis, and chemoresistance through its interactions with a diverse array of signaling pathways and microRNAs [41, 42]. Collectively, these examples illustrate the multifaceted roles of lncRNAs in cancer biology and underscore their potential as biomarkers and therapeutic targets.

NUTM2A-AS1 (NUT Family Member 2A–Antisense RNA 1) is a 3.7-kb long noncoding RNA that has been mapped to human chromosome 19. It has been localized to both the nucleus and the cytoplasm, as indicated by GeneCards and supported by functional studies, where it regulates gene expression during differentiation and post-transcriptional processes. NUTM2A-AS1 is an antisense long noncoding RNA transcribed from the opposite strand of the NUTM2A gene. While their genomic overlap implies potential cis-regulatory interactions, no direct regulatory effect between NUTM2A-AS1 and NUTM2A has been reported to date. Although most studies on NUTM2A-AS1 have focused on cancer, emerging evidence also implicates this lncRNA in inflammatory and degenerative diseases such as rheumatoid arthritis, pulpitis, and osteoarthritis [43–45]. In these contexts, NUTM2A-AS1 appears to contribute to inflammatory responses and cellular damage through mechanisms involving ceRNA-mediated regulation of pro-inflammatory signaling pathways. The present review is designed to systematically investigate the emerging role of NUTM2A-AS1 across different cancer types. By examining its molecular functions, interactions with regulatory networks, and contributions to tumorigenesis, this review aims to provide a comprehensive understanding of how alterations in NUTM2A-AS1 expression may influence cancer development and progression. The analysis presented herein is intended to elucidate the potential of NUTM2A-AS1 as a diagnostic biomarker and as a target for therapeutic intervention in oncology.

## Assessment of NUTM2A-AS1 expression levels and its association with clinical characteristics across different tumor types

Several studies have highlighted the significance of NUTM2A-AS1 in malignant tumors. This review explores the influence of NUTM2A-AS1 expression on various cancers (Table 1).

**Table 1:** NUTM2A-AS1 expression, functional role, and cell lines used in various cancers

Cancer type	Role	Expression status	Cell lines used
Gastric cancer	Oncogenic Oncogenic	Upregulated Upregulated	HGC-27, SNU-1 N87, AGS
Hepatocellular carcinoma	Oncogenic Oncogenic	Upregulated Upregulated	HCCLM3, Huh7 HepG2
Neuroblastoma	Oncogenic	Upregulated	SK-N-SH, SHSY-5Y
Colorectal cancer	Oncogenic	Upregulated	Not specified
Glioma	Oncogenic	Upregulated	U251, T98-G, A172, HEB
Lung adenocarcinoma	Oncogenic	Upregulated	NCI-H23, A549, BEAS2B
Prostate cancer	Oncogenic	Upregulated	RWPE-2, DU145, PC-3, 22RV1, C4-2B, LNCap
Renal cell carcinoma	Oncogenic	Upregulated	Not specified

### Gastric cancer

Gastric cancer (GC) is a highly lethal malignancy, ranking as the fifth most frequently diagnosed cancer and the fourth leading cause of cancer-related death globally, with a dismal 5-year survival rate below 10% [46, 47]. Despite advances in treatment—where surgery followed by adjuvant chemotherapy or chemoradiotherapy is the standard for eligible patients—approximately 60% of individuals experience recurrence or distant metastases after resection [48, 49]. Moreover, nearly one-third of GC patients present with peritoneal metastasis at diagnosis, and a marked gender disparity exists, with males exhibiting almost twice the incidence of females [5, 50].

Recently published research aimed to elucidate the molecular mechanism by which NUTM2A‑AS1 contributes to GC progression [51]. The investigators quantified mRNA and protein levels using RT‑qPCR and western blotting, respectively, and assessed functional outcomes—cell viability by MTT assay, invasion via Transwell assays, and drug resistance through both in vitro and in vivo approaches including xenograft experiments and analysis of The Cancer Genome Atlas (TCGA) datasets [51]. Mechanistically, NUTM2A‑AS1 was found to enhance cell viability, invasion, and chemoresistance, effects that were largely mitigated by miR‑376a. Further experiments demonstrated that miR‑376a negatively regulated TET1 and HIF‑1A (Hypoxia-Inducible Factor 1-alpha), with TET1 interacting with HIF‑1A to upregulate PD‑L1 levels. This study provides evidence that the NUTM2A‑AS1/miR‑376a/TET1/PD‑L1 axis plays a key role in GC tumorigenesis and suggests that combining conventional chemotherapy with immunotherapy could yield superior treatment outcomes.

A second study explored whether NUTM2A‑AS1 is involved in the inhibition of GC tumorigenesis by *matrine*, a compound known for its anti‑cancer properties. In this work, assays including MTT, colony formation, and TUNEL were employed to assess proliferation, viability, and apoptosis [52]. The authors also quantified levels of NUTM2A‑AS1, miR‑613, and VEGFA (Vascular Endothelial Growth Factor A), and evaluated oxidative stress parameters (reactive oxygen species, glutathione content, and superoxide dismutase activity). Knockdown of NUTM2A‑AS1 in N87 and AGS cell lines led to reduced viability and proliferation and increased apoptosis following *matrine* treatment, effects that were reversed upon inhibition of miR‑613 [52]. These results support the existence of a NUTM2A‑AS1/miR‑613/ROS/VEGFA regulatory axis that influences *matrine* resistance in GC.

### Hepatocellular carcinoma

Hepatocellular carcinoma (HCC) is the most common type of liver cancer, representing 80–90% of primary liver malignancies and ranking as the 6th most common cancer worldwide and the 4th leading cause of cancer-related death [53–55]. Despite improvements in early detection and treatment modalities such as radical surgeries, a significant number of patients are diagnosed at advanced stages, which contributes to high mortality rates [56]. Chronic liver inflammation from factors such as HBV, HCV, aflatoxin exposure, and alcohol consumption remains the strongest risk factor for HCC [57, 58].

**Fig. 1:**
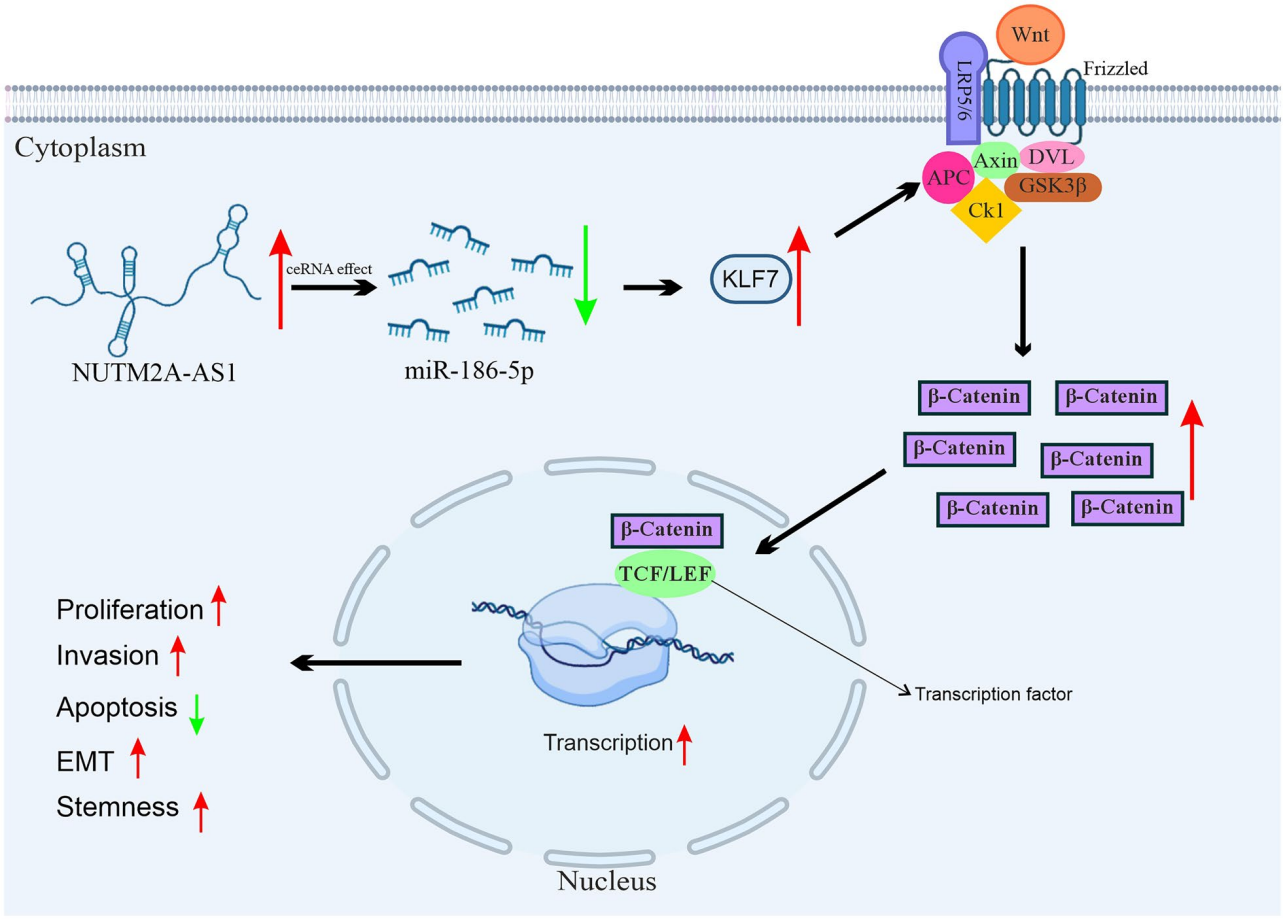
Schematic model of the NUTM2A-AS1/miR-186-5p/KLF7/Wnt/β catenin axis in hepatocellular carcinoma. Elevated NUTM2A-AS1 in HCC acts as a competing endogenous RNA (ceRNA) to sponge miR-186-5p, leading to upregulation of KLF7. KLF7 in turn contributes to the activation of the Wnt/β-catenin pathway. In the absence of Wnt ligand (Wnt OFF state), β-catenin is targeted for degradation by a destruction complex composed of Axin, APC, GSK-3, and CK1. Upon Wnt activation (Wnt ON state), this complex is inhibited, allowing β-catenin to accumulate and translocate into the nucleus. There, β-catenin cooperates with LEF/TCF transcription factors to promote the transcription of Wnt target genes. This pathway drives key oncogenic processes, including proliferation, invasion, epithelial–mesenchymal transition (EMT), and stemness in hepatocellular carcinoma

One study investigated the role of NUTM2A‑AS1 in HCC by comparing its expression in tumor tissues and adjacent normal tissues [59]. Functional assays in HCC cell lines (HCCLM3 and Huh7) revealed that elevated NUTM2A‑AS1 levels were associated with larger tumor size, advanced stage, and lymph node metastasis. Mechanistic investigations uncovered that NUTM2A‑AS1 acts as a competitive endogenous RNA (ceRNA) for miR‑186‑5p, thereby upregulating the transcription factor KLF7. The downstream activation of the Wnt/β‑catenin signaling pathway resulted in enhanced proliferation, invasion, epithelial–mesenchymal transition (EMT), and stemness, while apoptosis was concurrently suppressed [59]. Rescue experiments further confirmed that silencing miR‑186‑5p or overexpressing KLF7 could reverse the anti‑tumor effects mediated by NUTM2A‑AS1 knockdown (Fig. 1).

A parallel study utilized bioinformatics approaches alongside in vitro experiments to assess the influence of curcumin on key lncRNAs and miRNAs implicated in HCC. The study identified NUTM2A‑AS1 (along with HCG18) as one of the lncRNAs interacting with a broad network of miRNAs [60]. Treatment of HepG2 cells with curcumin (at half‑inhibitory concentrations) led to significant upregulation of tumor‑suppressive miRNAs (miR‑195, miR‑15a/16, and miR‑424) and concurrent downregulation of NUTM2A‑AS1, HCG18, and miR‑15b‑5p. These changes correlated with alterations in oxidative stress parameters, suggesting that curcumin may exert its anti‑cancer effects through modulation of the NUTM2A‑AS1/miRNA axis [60]. While these findings provide novel insights into the molecular underpinnings of curcumin’s anti‑tumor activity, additional in vivo studies are necessary to substantiate these observations.

### Neuroblastoma

Neuroblastoma (NB) is the most common extracranial solid tumor in children, accounting for 8–10% of pediatric cancers and approximately 15% of cancer-related deaths in this population [61–63]. While multimodal treatment strategies have improved survival for high-risk cases from 15% to nearly 50%, a significant portion of patients remain refractory to first-line therapies [64, 65]. The disease is characterized by considerable clinical heterogeneity, influenced by factors such as age at diagnosis, tumor localization, and genetic markers like MYCN amplification [66, 67].

Recent work demonstrated that NUTM2A‑AS1 expression is elevated in cisplatin‑resistant NB cells in both a time‑ and dose‑dependent manner [68]. Functional knockdown of NUTM2A‑AS1 not only increased the sensitivity of NB cells to cisplatin but also reduced their metastatic potential. Mechanistically, NUTM2A‑AS1 was found to stabilize B7 homolog 3 protein (B7‑H3), an immune checkpoint protein, by inhibiting its degradation via the ubiquitin‑proteasome pathway. Bioinformatics analysis and subsequent experimental validation (including ChIP and luciferase reporter assays) revealed that NR1D1 acts as a transcriptional activator of NUTM2A‑AS1 [68]. Chromatin immunoprecipitation (ChIP) was performed using the Merck Millipore ChIP Assay Kit (Protein A agarose/Salmon Sperm DNA, Cat. #16–157) following the manufacturer’s protocol. Immunoprecipitation was carried out using an anti-NR1D1 antibody, and qPCR was subsequently conducted to assess enrichment at the NUTM2A-AS1 promoter region, specifically targeting the proximal ~ 1 kb upstream of the transcription start site. This NR1D1/NUTM2A‑AS1/B7‑H3 axis provides a compelling explanation for cisplatin resistance and immune evasion in NB, offering a potential target for future therapeutic intervention.

### Colorectal cancer

Colorectal cancer (CRC) is a major global health burden, being the third most commonly diagnosed cancer in the United States and worldwide, with over 1.9 million new cases and 0.9 million deaths reported in 2020 [69, 70]. Although advances in screening methods—such as colonoscopy and fecal tests—have led to a decline in overall colorectal cancer incidence, a significant number of patients are still diagnosed with metastatic disease or develop metastases over time, highlighting the limitations of screening alone in reducing late-stage disease burden; notably, 20% of patients present with synchronous metastases at diagnosis, and an additional 40% develop metastases during follow-up [71, 72]. The etiology of CRC is significantly influenced by modifiable risk factors such as smoking, unhealthy diet, high alcohol intake, physical inactivity, and obesity [73].

In one study, quantitative real‑time PCR analysis revealed that NUTM2A‑AS1 is markedly elevated in CRC cell lines [74]. Functional assays—including CCK‑8 (Cell Counting Kit-8) for proliferation and flow cytometry for apoptosis—demonstrated that silencing of NUTM2A‑AS1 resulted in reduced proliferation and increased apoptotic rates. Mechanistically, NUTM2A‑AS1 was shown to be transcriptionally activated by histone H3K27ac at its promoter and to act as a sponge for miR‑126‑5p, leading to the upregulation of FAM3C (Family With Sequence Similarity 3 Member C) expression [74]. The study posits that NUTM2A‑AS1 functions as an oncogenic lncRNA in CRC, representing a potential novel therapeutic target for intervention.

### Glioma

Gliomas, particularly glioblastoma multiforme (GBM), represent some of the most aggressive and deadly brain tumors, with median survival times ranging between 12.5 and 15 months and 5-year survival rates as low as 10% [75, 76]. GBM accounts for approximately 80% of intracranial malignancies in adults and is notorious for its genetic heterogeneity and diffuse infiltrative nature [77–79]. Standard treatment regimens—comprising surgical resection, chemotherapy, and radiotherapy—offer only limited benefits, which underscores the need for novel molecular targets [80].

A study investigating NUTM2A‑AS1 in glioma cell lines (U251 and A172) found that its expression was significantly elevated compared to non‑tumor cells, while miR‑376a‑3p levels were concomitantly reduced [81]. Using StarBase software and dual‑luciferase reporter assays, the study identified miR‑376a‑3p as a direct target of NUTM2A‑AS1. Functional assays (MTT for proliferation and flow cytometry for apoptosis) indicated that silencing NUTM2A‑AS1 enhanced miR‑376a‑3p expression, thereby reducing proliferation and promoting apoptosis. Importantly, YAP1 (Yes-associated protein 1) was validated as a downstream target of miR‑376a‑3p, with its expression inversely correlated with miR‑376a‑3p levels [81]. Although the data support a model in which the NUTM2A‑AS1/miR‑376a‑3p/YAP1 axis contributes to glioma progression, further in vivo studies are required to fully elucidate this regulatory network. Moreover, potential regulation of YAP1 by additional miRNAs suggests that this axis may be part of a broader, more complex regulatory mechanism.

### Lung adenocarcinoma

Lung adenocarcinoma (LUAD) is the most common subtype of lung cancer and a leading cause of cancer-related deaths globally [82, 83]. Despite the advent of low-dose computed tomography (CT) that has enabled early detection—yielding impressive 5-year survival rates of up to 94.9% in stage I LUAD—the prognosis for advanced stages remains grim, with a 5-year survival rate of approximately 50% [84, 85]. LUAD is characterized by substantial molecular heterogeneity and complex alterations in the tumor microenvironment, including prevalent KRAS mutations linked to smoking [86, 87].

In LUAD, the oncogenic potential of NUTM2A‑AS1 has been investigated with respect to its modulation of apoptosis and cell viability. The study employed RT‑qPCR to reveal significant upregulation of NUTM2A‑AS1 and downregulation of miR‑590‑5p in LUAD cell lines compared with normal epithelial cells [88]. Functional experiments (MTT assays and flow cytometry) demonstrated that knockdown of NUTM2A‑AS1 in NCI‑H23 cells resulted in decreased viability and increased apoptosis. Further mechanistic insights were provided by dual‑luciferase reporter assays, which confirmed miR‑590‑5p as a direct target of NUTM2A‑AS1. In addition, METTL3, a methyltransferase implicated in RNA modification, was shown to be significantly upregulated in LUAD cells and to be directly inhibited by miR‑590‑5p [88]. Thus, the NUTM2A‑AS1/miR‑590‑5p/METTL3 axis appears to play a crucial role in LUAD progression, offering a potential new avenue for therapeutic intervention.

### Other cancers

Prostate cancer (PCa) is one of the most frequently diagnosed malignancies in men, with a global incidence exceeding 1.4 million new cases and a significant mortality burden, especially among older men [5, 89]. Over 95% of PCa cases are adenocarcinomas, predominantly originating from the peripheral zone of the prostate, and exhibit notable racial disparities, with higher incidence and mortality among African American and Caribbean men [90, 91]. Diagnosis typically follows an elevated prostate-specific antigen (PSA) screening and is confirmed through biopsy and Gleason grading, which guide treatment decisions [92, 93]. In PCa, the role of NUTM2A‑AS1 has been evaluated in relation to cancer stem cell (CSC)‑like traits and tumor progression. Expression analyses in PCa patient samples and the DU145 cell line indicated elevated levels of NUTM2A‑AS1 and PRMT5, with a concomitant reduction in miR‑376a‑3p. Functional assays (cell counting kit‑8, Transwell migration/invasion assays, apoptosis assays, and tumor sphere formation) demonstrated that both inhibition of NUTM2A‑AS1 and overexpression of miR‑376a‑3p markedly reduced cell proliferation and CSC‑like characteristics [94]. Mechanistically, NUTM2A‑AS1 acts as a ceRNA for miR‑376a‑3p, thereby modulating PRMT5 expression. The study concludes that the NUTM2A‑AS1/miR‑376a‑3p/PRMT5 axis is critical for the maintenance of oncogenic traits in PCa, highlighting its potential as a target for novel therapeutic strategies.

Renal cell carcinoma (RCC) comprises about 3% of adult cancers and is a heterogeneous disease, with clear cell RCC (ccRCC) accounting for 70–80% of cases [95, 96]. ccRCC is marked by inactivation of the von-Hippel-Lindau (VHL) gene, leading to hyperactivation of hypoxia-inducible factors and overexpression of genes involved in angiogenesis, metabolism, and cell cycle regulation, which contribute to its aggressive behavior; nearly 30% of patients develop metastases [97, 98]. In the United States, RCC has a lifetime prevalence of 2.3% in men and 1.3% in women, with around 81,800 new cases reported in 2023 [99]. An additional study focused on renal cell carcinoma (RCC) sought to identify lncRNA‐based prognostic signatures for high‑grade and high‑stage disease [100]. Utilizing data from the Surveillance, Epidemiology, and End Results (SEER) database and TCGA, the researchers identified several independent prognostic factors, including pathological stage and histological grade. Through Lasso analysis and Cox regression modeling, a 9‑lncRNA signature was established that included NUTM2A‑AS1. Kaplan–Meier survival analysis demonstrated that patients with stage IV, grade G4 RCC and high risk scores had significantly poorer overall survival. Pathway enrichment analysis implicated these lncRNAs in cell division, cell cycle regulation, and DNA damage response [100]. Although NUTM2A-AS1 is incorporated into a prognostic lncRNA signature for RCC, its precise mechanistic role in disease progression remains unclear, as no direct downstream regulatory axis has been reported to date.

## Potential underlying NUTM2A-AS1 functions

Accumulating evidence indicates that NUTM2A-AS1 is a multifunctional long noncoding RNA whose dysregulation contributes to various aspects of tumor biology. Several interrelated molecular mechanisms have been proposed to explain its oncogenic roles:

### Acting as a competing endogenous RNA (ceRNA)

**Fig. 2:**
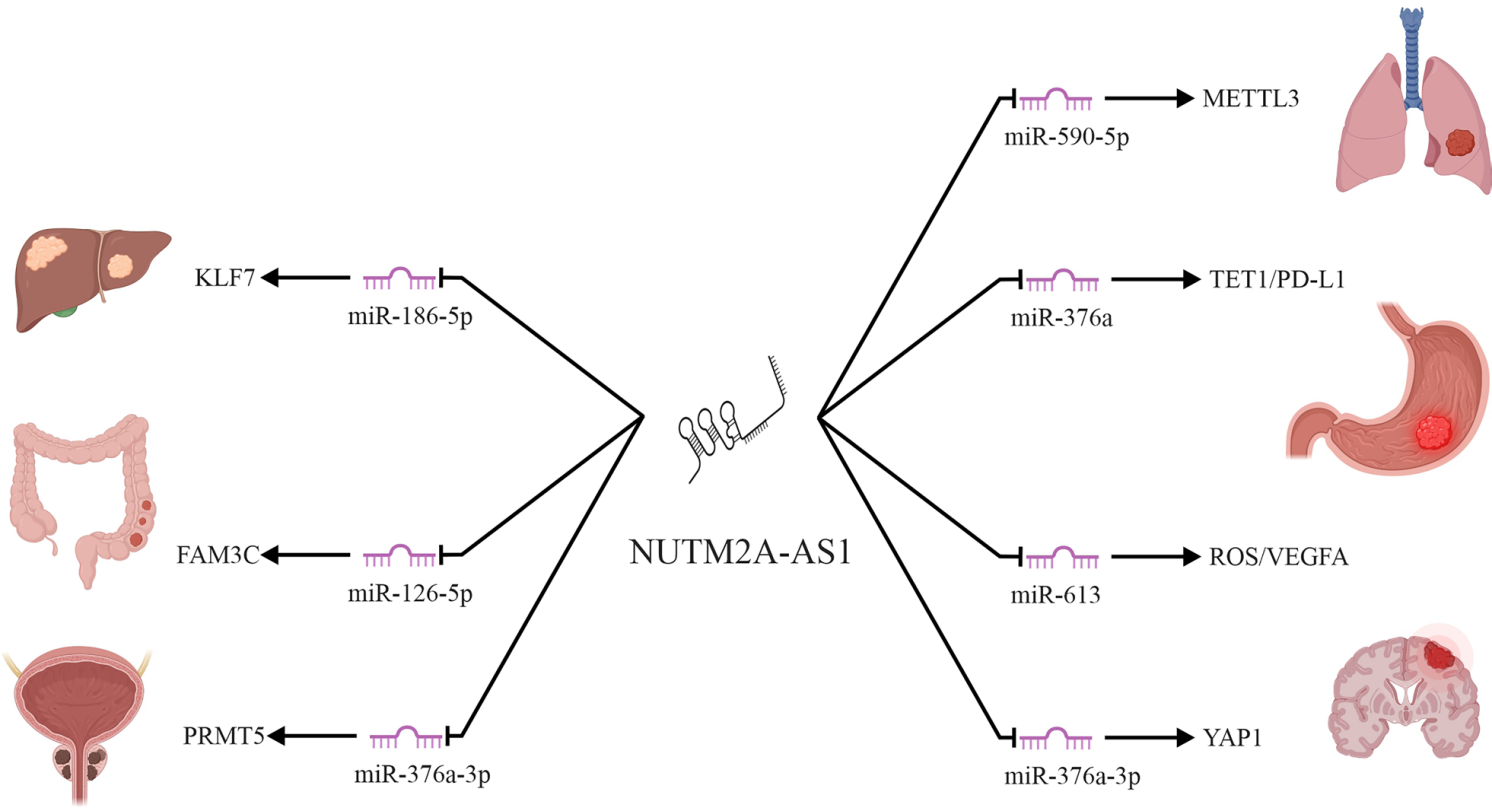
Schematic diagram of the proposed ceRNA regulatory network involving NUTM2A-AS1, including interactions with miRNAs and mRNAs in various cancers

The ceRNA mechanism describes how various RNAs, including lncRNAs, pseudogenes, and circRNAs, act as molecular sponges for miRNAs, thereby modulating mRNA expression [101, 102]. First proposed by Salmena et al. in 2011, this hypothesis suggests that ceRNAs compete for miRNA binding sites, influencing downstream gene regulation [103]. Long noncoding RNAs frequently regulate gene expression by acting as competing endogenous RNAs (ceRNAs), binding to microRNAs and relieving their suppression of target mRNAs [104, 105]. This mechanism allows lncRNAs to indirectly modulate the levels of critical proteins involved in tumorigenesis. By sequestering specific microRNAs, these molecules contribute to diverse cellular processes such as proliferation, invasion, and resistance to therapy [21, 106]. NUTM2A-AS1 exemplifies this function by targeting distinct microRNAs across different cancers (Table 2). In gastric cancer, NUTM2A-AS1 sponges miR-376a, which normally suppresses TET1 and HIF-1A; the resulting increase in these proteins subsequently elevates PD-L1 expression, thereby promoting cell viability, invasion, and chemoresistance [51]. Similarly, in hepatocellular carcinoma, it binds miR-186-5p to derepress KLF7 and activate the Wnt/β catenin pathway, enhancing proliferation and EMT [51]. In colorectal cancer, transcriptional activation via H3K27 acetylation permits NUTM2A-AS1 to sequester miR-126-5p, leading to upregulation of FAM3C [74], while in glioma, its interaction with miR-376a-3p regulates YAP1 expression [81]. The ceRNA mechanism is also evident in lung adenocarcinoma through the miR-590-5p/METTL3 axis [88] and in prostate cancer via modulation of PRMT5 through miR-376a-3p [94] (Fig. 2).

**Table 2:** Downstream regulatory factors modulated by NUTM2A-AS1 in various cancers and experimental methodologies

Cancer type	Downstream regulatory factors	Methodologies	Year	Refs
Gastric cancer	miR-376a/TET1/HIF-1A/PD-L1 miR-613/ROS/VEGFA	RT-qPCR, Western blot, MTT assay, Transwell invasion assay, dual-luciferase reporter assay, RNA pull-down, RNA immunoprecipitation, xenograft studies, TCGA analysis	2020 2022	[51] [52]
Hepatocellular carcinoma	miR-186-5p/KLF7	RT-qPCR, Western blot, dual-luciferase reporter assay, bioinformatics analysis	2022	[59]
Neuroblastoma	NR1D1/NUTM2A-AS1/B7-H3	RT-qPCR, ChIP assay, luciferase reporter assay, functional knockdown, cell viability and apoptosis assays	2024	[68]
Colorectal cancer	miR-126-5p/FAM3C	RT-qPCR, CCK-8 assay, flow cytometry, dual-luciferase reporter assay	2024	[74]
Glioma	miR-376a-3p/YAP1	RT-qPCR, MTT assay, flow cytometry, StarBase analysis, dual-luciferase reporter assay	2024	[81]
Lung adenocarcinoma	miR-590-5p/METTL3	RT-qPCR, MTT assay, flow cytometry, dual-luciferase reporter assay	2021	[88]
Prostate cancer	miR-376a-3p/PRMT5	Cell counting kit-8 assay, Transwell migration/invasion assays, apoptosis assays, tumor sphere formation assay	2024	[94]
Renal cell carcinoma	Not reported	Data mining from SEER and TCGA, Lasso analysis, Cox regression modeling, Kaplan–Meier survival analysis	2021	[100]

### Regulation of oxidative stress and apoptosis

Cellular redox balance is a critical determinant of cancer cell survival and response to therapy [107, 108]. Modulation of reactive oxygen species (ROS) and antioxidant defenses can influence apoptosis, proliferation, and resistance to chemotherapeutics [109]. lncRNAs may alter oxidative stress responses by affecting the expression of key regulatory molecules and enzymes. In gastric cancer, NUTM2A-AS1 contributes to *matrine* resistance by forming a regulatory axis with miR-613 and VEGFA, where its knockdown decreases cell viability, reduces proliferation, and enhances apoptosis in tandem with altered ROS, glutathione levels, and superoxide dismutase activity [52]. A similar paradigm is observed in hepatocellular carcinoma, where curcumin-induced downregulation of NUTM2A-AS1 correlates with increased levels of tumor-suppressive miRNAs and shifts in oxidative stress parameters, collectively impeding tumor progression [60].

### Modulation of immune checkpoints and drug resistance

The tumor microenvironment and immune evasion are central to cancer progression and therapy failure. lncRNAs can influence the expression and stability of immune checkpoint proteins, thereby modulating the anti-tumor immune response [110–112]. NUTM2A-AS1 is implicated in these processes by regulating key immune modulators. In gastric cancer, by indirectly enhancing PD-L1 expression through the miR-376a/TET1/HIF-1A pathway, NUTM2A-AS1 promotes immune escape and chemoresistance [51]. In neuroblastoma, elevated NUTM2A-AS1 stabilizes B7-H3 by inhibiting its ubiquitin mediated degradation, a process further driven by its transcriptional activation via NR1D1 [68]. These axes underscore its dual role in fostering drug resistance and immune evasion.

### Epigenetic and transcriptional regulation

Epigenetic modifications can govern lncRNA expression and, in turn, affect downstream signaling pathways crucial for tumorigenesis [113]. Histone modifications such as H3K27 acetylation are known to activate gene transcription and have been linked to the upregulation of oncogenic lncRNAs [114]. In colorectal cancer, for example, H3K27ac enrichment at the NUTM2A-AS1 promoter is pivotal for its transcriptional activation, thereby enabling its function as a ceRNA for miR-126-5p and the consequent upregulation of FAM3C [74]. This layer of epigenetic control further emphasizes the complex regulatory networks in which NUTM2A-AS1 is embedded across different cancer types.

## Conclusion

In summary, the accumulated research on NUTM2A-AS1 underscores its multifaceted role as an oncogenic long noncoding RNA (lncRNA) in cancer. Across diverse malignancies—including gastric cancer, hepatocellular carcinoma, neuroblastoma, colorectal cancer, glioma, lung adenocarcinoma, and prostate cancer—NUTM2A-AS1 has emerged as a critical regulator of tumor progression. Predominantly operating as a competing endogenous RNA (ceRNA), NUTM2A-AS1 sequesters specific microRNAs such as miR-376a, miR-613, miR-186-5p, miR-126-5p, and miR-590-5p. This sequestration effectively derepresses downstream targets including TET1, HIF-1A, VEGFA, KLF7, YAP1, METTL3, and PRMT5, which in turn contribute to enhanced cell proliferation, invasion, stemness, immune evasion, and drug resistance. Notably, transcriptional activation of NUTM2A-AS1 via H3K27 acetylation has been reported in colorectal cancer. While similar epigenetic regulation has not been confirmed in other cancers, this finding suggests potential upstream chromatin-based control that warrants further investigation.

The biological and clinical significance of these findings is considerable. As an important component within cancer-related ceRNA networks, NUTM2A-AS1 not only facilitates tumorigenesis but also holds promise as a biomarker for diagnosis, prognosis, and therapeutic stratification. The observed correlations between high NUTM2A-AS1 expression and advanced tumor stage or poor clinical outcome across several cancer types suggest that it may serve as a valuable prognostic indicator. Moreover, its involvement in drug resistance pathways, such as the modulation of PD-L1 expression in gastric cancer and the stabilization of immune checkpoint proteins in neuroblastoma, positions NUTM2A-AS1 as a potential target for novel therapeutic interventions.

Emerging technologies such as antisense oligonucleotides (ASOs), CRISPR-dCas9 epigenetic editing, and siRNA delivery platforms hold promise for targeting NUTM2A-AS1 in preclinical models. However, experimental validation of these approaches specific to NUTM2A-AS1 remains a critical next step.

Despite these advances, current research on NUTM2A-AS1 is not without limitations. Many studies have predominantly relied on in vitro models, and while some in vivo validations exist, further animal studies and clinical trials are needed to confirm these mechanisms and to explore the translational potential of targeting NUTM2A-AS1. In addition, the complexity of its ceRNA interactions, along with variability across different tumor types, calls for a more nuanced understanding of its role in cancer heterogeneity.

In conclusion, while our current understanding of NUTM2A-AS1 as a key ceRNA in cancer has substantially advanced, ongoing and future studies are essential to fully elucidate its molecular mechanisms and to harness its potential in improving cancer diagnosis, prognosis, and treatment.

## Data Availability

No datasets were generated or analysed during the current study.

## Abbreviations

GC: Gastric cancer
HCC: Hepatocellular carcinoma
NB: Neuroblastoma
CRC: Colorectal cancer
GBM: Glioblastoma
LUAD: Lung adenocarcinoma
PCa: Prostate cancer
RCC: Renal cell carcinoma
miRNAs: MicroRNAs
lncRNAs: Long noncoding RNAs
circRNAs: Circular RNAs
ceRNA: Competitive endogenous RNA
TCGA: The Cancer Genome Atlas
EMT: Epithelial–mesenchymal transition
